# Co-Production of Fungal Biomass Derived Constituents and Ethanol from Citrus Wastes Free Sugars without Auxiliary Nutrients in Airlift Bioreactor

**DOI:** 10.3390/ijms17030302

**Published:** 2016-02-26

**Authors:** Behzad Satari, Keikhosro Karimi, Mohammad J. Taherzadeh, Akram Zamani

**Affiliations:** 1 Swedish Centre for Resource Recovery, University of Borås, 50190 Borås, Sweden; B.Satari@ce.iut.ac.ir (B.S.); Mohammad.Taherzadeh@hb.se (M.J.T.); 2 Department of Chemical Engineering, Isfahan University of Technology, Isfahan 84156-83111, Iran; Karimi@cc.iut.ac.ir

**Keywords:** chitosan, citrus waste, ethanol, *Mucor indicus*, oil, pellet, protein, *Rhizopus oryzae*

## Abstract

The potential of two zygomycetes fungi, *Mucor indicus* and *Rhizopus oryzae*, in assimilating citrus waste free sugars (CWFS) and producing fungal chitosan, oil, and protein as well as ethanol was investigated. Extraction of free sugars from citrus waste can reduce its environmental impact by decreasing the possibility of wild microorganisms growth and formation of bad odors, a typical problem facing the citrus industries. A total sugar concentration of 25.1 g/L was obtained by water extraction of citrus waste at room temperature, used for fungal cultivation in shake flasks and airlift bioreactor with no additional nutrients. In shake flasks cultivations, the fungi were only able to assimilate glucose, while fructose remained almost intact. In contrast, the cultivation of *M. indicus* and *R. oryzae* in the four-liter airlift bioreactor resulted in the consumption of almost all sugars and production of 250 and 280 g fungal biomass per kg of consumed sugar, respectively. These biomasses correspondingly contained 40% and 51% protein and 9.8% and 4.4% oil. Furthermore, the fungal cell walls, obtained after removing the alkali soluble fraction of the fungi, contained 0.61 and 0.69 g chitin and chitosan per g of cell wall for *M. indicus* and *R. oryzae*, respectively. Moreover, the maximum ethanol yield of 36% and 18% was obtained from *M. indicus* and *R. oryzae*, respectively. Furthermore, that *M. indicus* grew as clump mycelia in the airlift bioreactor, while *R. oryzae* formed spherical suspended pellets, is a promising feature towards industrialization of the process.

## 1. Introduction

The world production of citrus fruits in 2010/11 was over 115 million tons, of which almost 70 million tons was orange [1]. Over 23 million tons of orange was utilized for processing, producing 50%–60% organic waste [1]. Considering the potential environmental problems caused by this huge quantity of organic chemicals, efficient citrus waste treatment is vital for citrus industries.

A minor part of citrus wastes (CW) is used to industrially produce fiber and food ingredients, e.g., pectin, mucilage, and flavonoids [2]. A part of CW is also used as a traditional cattle feed in the form of dried pellet or dehydrated paste. However, a major part is still disposed of into the environment. Recently, it was suggested to use orange peels for the production of biofuels, limonene, and other co-products [3,4,5]. The carbohydrate polymers in the CW, *i.e.*, cellulose and hemicelluloses, can potentially be converted to fermentable sugars in order to produce different metabolites [6,7,8]. Besides the carbohydrate polymers, CW contain considerable amounts of free mono- and di-saccharides, which remain intact after juice extraction. The presence of these free sugars in CW can cause problems associated with the extracted chemicals purity, e.g., pectin, and wastewater treatment of the extraction processes. Furthermore, the presence of free sugars in the citrus wastes is accompanied with the possibility of growth of pathogenic bacteria and fungi as well as the formation of bad odors. However, the free sugars can be extracted and used as carbon- and nutrient-rich medium for fungal cultivation under controlled conditions.

*Mucor indicus* and *Rhizopus oryzae*, two edible non-pathogenic strains of zygomycetes fungi, are classified as ethanol-producing microorganisms with several advantages compared to *Saccharomyces cerevisiae* [9,10]. Beside a high ethanol yield, the biomass of these fungi contains valuable materials, including chitosan, protein, and fungal-oil [11]. It has also been reported that the filamentous fungus *R. oryzae* can form spherical pellets under controlled conditions, which is the desired morphology for industrial applications [12,13]. Fungal growth in pellet form has many advantages, e.g., facilitating aeration, agitation, and heat transfer, reducing the culture viscosity and probability of wrapping around probes and impellers. This morphology is also suitable for reusing the biomass [14].

A variety of carbohydrates, including free sugars and hydrolysates of starch and lignocellulosic materials, have been used as a carbon source for the cultivation of these strains [15,16]. However, in a majority of the studies, the carbohydrate solutions have been supplemented with appreciable amounts of supplementary nutrients, e.g., salts, trace metals, vitamins, and yeast extract [17,18]. The requirement of such nutrients makes the commercial application of these fungi impractical. The CW water-soluble extracts are supposed to contain free sugars as well as complementary components necessary for cell growth.

The purpose of this study was to investigate the possibility of an efficient and practical solution for reducing the problem of citrus waste using industrialization capability. Water extraction was applied to extract the free sugars and other water soluble materials of the CW. The sugars were then fermented using the filamentous fungi *M. indicus* and *R. oryzae*. The obtained fungal biomass was analyzed for oil, chitosan, and protein contents.

## 2. Results

**Figure 1:**
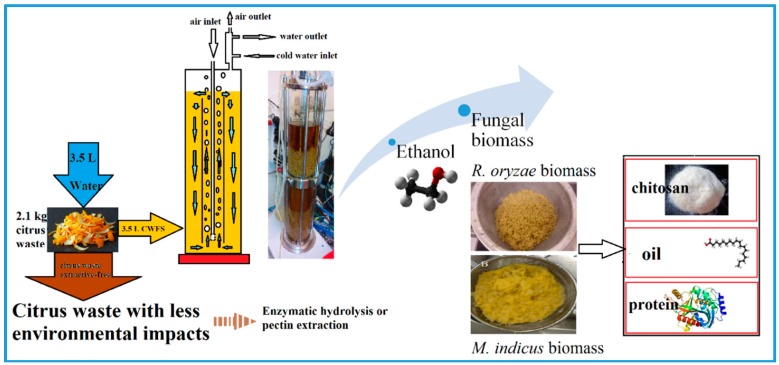
Lab-scale study on fungal conversion of citrus waste sugars.

Fungal biomass and ethanol production from citrus wastes (CW) with minimal auxiliary nutrients usage was the main focus of the present study. The fungal biomass and its derivatives contain different value-added chemicals. Schematic of the process, including extraction, cultivation in an airlift bioreactor, and biomass harvesting, is shown in Figure 1.

The raw CW, as received, had a dry weight of 20.5% where water-soluble extracts made up 56.1% of the dry weight.

### 2.1. Extraction of Free Sugars

The aim of this step was to maximize the recovery of free sugars. The preliminary experiments showed that temperature had no significant effect on the sugar recovery. In addition, no considerable difference was observed in the recovery for 15 min and 2 h extraction. Table 1 shows four experiments with two levels of CW to water ratio and two types of mixing. Accordingly, 5:3 (L:kg) water to CW ratio exhibited the highest recovery. Since water bath mixing was by far easier and less energy consuming than blender mixing, it was selected as the best method for extraction of CWFS (experiment number 4). The concentration of the sugars extracted by this method was 11.1 ± 0.5 g/L glucose, 10.6 ± 0.5 g/L fructose, and 3.4 ± 0.6 g/L sucrose. Total solid (TS %) of the extract and ash content of TS were 2.7% and 14.5% (on TS basis), respectively.

### 2.2. Fungal Growth in Shake Flasks

The purpose of fungal cultivation in shake flasks was to examine the impact of complementary nutrients on sugar assimilation rate by the fungi for possible use in a bench scale airlift bioreactor. The strategy was to supplement CWFS medium with all the nutrients (including yeast extract, ammonium sulphate, dihydrogen potassium phosphate, magnesium sulphate, and calcium chloride) as well as media with only phosphate and without any nutrient.

**Figure 2:**
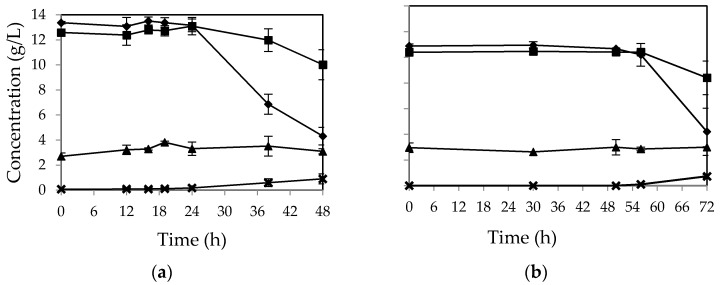
Concentration of (▲) sucrose, (♦) glucose, (■) fructose, and (×) ethanol in cultivation of (**a**) *M. indicus* and (**b**) *R. oryzae* on CWFS without any nutrient in baffled shake flasks. Data are means ± SD, *n* = 2.

In the absence of all the nutrients, over 67 percent of initial glucose was still assimilated after 48 h by *M. indicus* (Figure 2a). In contrast, fructose concentration was not consumed without nutrient addition. For *R. oryzae*, no considerable change in sugar concentration was observed after 48 h (Figure 2b). The fungus started to assimilate the sugars at the third day (over 60% of the sugars). However, the fructose uptake was still lower than that of glucose. The overall biomass yield for *M. indicus* and *R. oryzae* was 0.36 and 0.33 *g/g* of sugars consumed, respectively.

**Figure 3:**
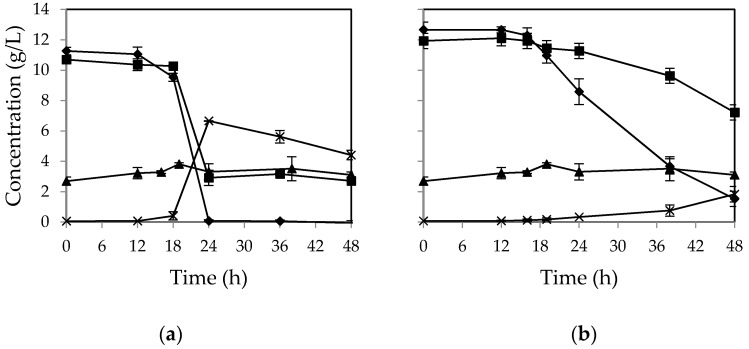
Concentration of (▲) sucrose, (♦) glucose, (■) fructose, and (×) ethanol in (**a**) CWFS supplemented with full nutrients and (**b**) CWFS supplemented with KH_2_PO_4_ in baffled shake flasks during the growth of *M. indicus*. Data are averages of two replicates ± SD.

Figure 3 shows the sugar consumption and ethanol production profiles during the cultivation of *M. indicus* on CWFS supplemented with different nutrients. In presence of all the nutrients, glucose was completely consumed during the first 24 h. Fructose consumption was started after glucose consumption, and the uptake rate was higher compared to the nutrient-free medium. A comparable phenomenon was observed when the sugar solution was supplemented with only phosphate (Figure 3b). No change in sucrose concentration was observed during cultivation; however, sucrose was partially hydrolyzed to glucose and fructose during autoclaving, which had a low pH (pH~4.1). The maximum ethanol yield was obtained after 24 h from *M. indicus* and nutrient-rich medium, which was 0.35 *g*/*g* of consumed sugars (corresponds to 69% ethanol theoretical yield, Figure 3a). The biomass yield was 0.39 and 0.42 *g*/*g* of consumed sugars for nutrient-rich and phosphate-rich medium, respectively.

Although the sugar assimilation patterns were rather different in the case of nutrient-rich and nutrient-free medium, improvement of sugar uptake by aeration was investigated in bench scale airlift reactors. The nutrients rich medium in airlift reactor was also tested for *M. indicus* for comparison.

### 2.3. Cultivation of “M. indicus” in Airlift Bioreactor with All Nutrients

**Figure 4:**
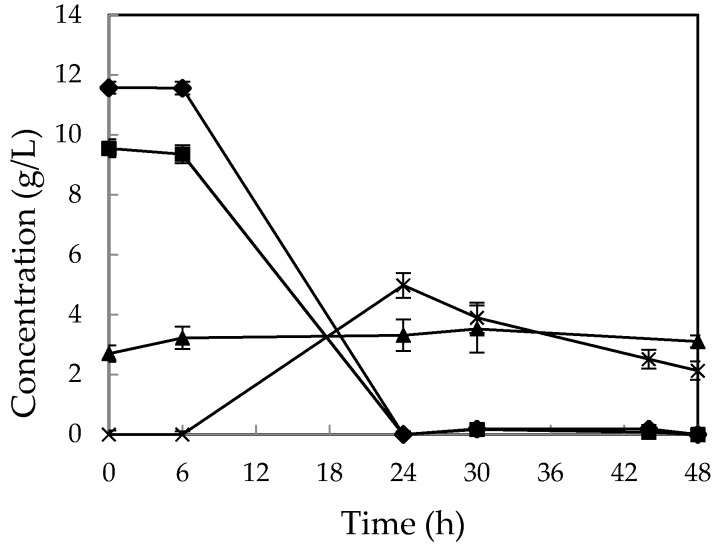
Concentration of (▲) sucrose, (♦) glucose, (■) fructose, and (×) ethanol in the cultivation of CWFS supplemented with all required nutrients in a 4-L airlift bioreactor using *M. indicus*. Data are means ± SD, *n* = 2.

The same sugar consumption patterns were observed for *M. indicus* in airlift reactor and shake flasks when CWFS solution was supplemented with all nutrients. After 24 h, all the sugars were consumed and maximum ethanol yield of 0.24 *g*/*g* on consumed sugars was obtained (Figure 4). Although ethanol yield was less than the same case in shake flasks, biomass yield was much higher. A biomass concentration of 13.97 g/L equivalent to 0.66 *g*/*g* consumed sugars was achieved after 48 h.

### 2.4. Cultivation of “M. indicus” and “R. oryzae” in Airlift Bioreactor without Any Nutrients

In order to investigate the impact of aeration on cell propagation in the absence of any nutrients, the fungi were cultivated on CWFS solution in airlift bioreactor. Since the fungi are not able to ferment sucrose [11], this disaccharide was hydrolyzed to glucose and fructose by invertase in the beginning of cultivations. 

**Figure 5:**
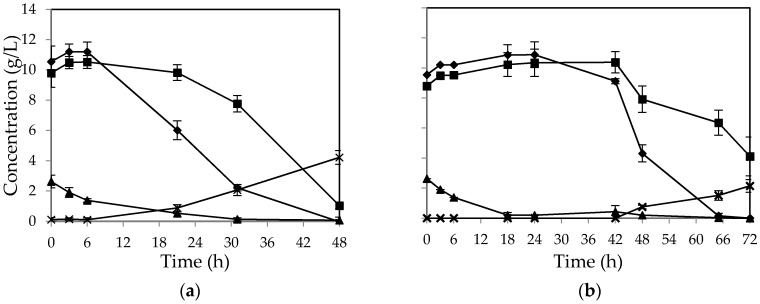
Concentration of (▲) sucrose, (♦) glucose, (■) fructose, and (×) ethanol in cultivation of (**a**) *M. indicus* and (**b**) *R. oryzae* in a 4-L airlift bioreactor on CWFS without nutrients supplementation. Data are means ± SD, *n* = 3.

Figure 5 shows the sugar consumption profile in airlift bioreactor for *M. indicus* (a) and *R. oryzae* (b). The results showed that fructose was consumed after glucose consumption. By depletion of glucose, the fructose consumption rate was increased. This behavior was similar to cultivation in shake flasks and nutrient-rich medium in the airlift reactor. Over 90 percent of initial sucrose was hydrolyzed during the first 18 h. However, sugar consumption patterns for the two fungi were different. The lag phase for *R. oryzae* was much longer in comparison with *M. indicus*, where glucose consumption was started for *R. oryzae* after 42 h of cultivation (Figure 5b). However, during the third day of cultivation a major reduction in sugar concentration occurred for this fungus. In contrast, for the *M. indicus*, the sugar concentrations were reduced gradually during the course of fermentation. Maximum ethanol production yield was 36 and 18 percent of theoretical yield for *M. indicus* and *R. oryzae,* respectively.

For both fungi, glucose was consumed completely during the cultivation. However, fructose concentration at the end was 1.0 and 4.1 g/L for *M. indicus* and *R. oryzae*, respectively. The biomass yields for *M. indicus* and *R. oryzae* were 0.25 and 0.28 *g*/*g* on consumed sugars, respectively (Table 2).

### 2.5. Fungal Morphology in Airlift Reactors

**Figure 6:**
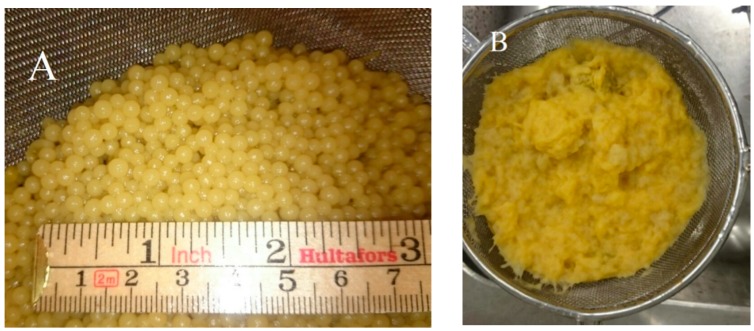
(**A**) Pellet type growth by *R. oryzae* and (**B**) mycelial type growth by *M. indicus* in airlift bioreactor on CWFS without any nutrients supplementation. The photos are taken after 72 and 48 h cultivation, respectively.

**Figure 7:**
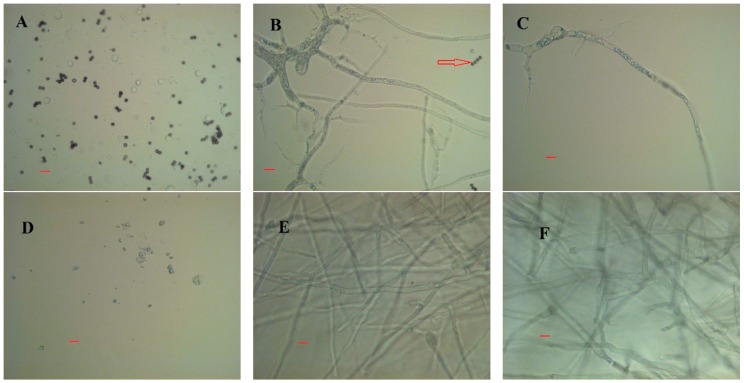
Microscopic morphology of *M. indicus* in airlift bioreactor after cultivation for (**A**) 0 h, (**B**) 24 h, and (**C**) 48 h. The **D**–**F** pictures belonged to *R. oryzae* growth (**D**) at the beginning, (**E**) after 48 h, and (**F**) 72 h. Both fungi were cultivated on CWFS in airlift reactors with no additional nutrients. The bars correspond to 20 μm.

Visual observations showed distinct differences in macromorphology of the two fungi grown in CWFS without auxiliary nutrients. *M. indicus* grew as freely dispersed mycelia, while *R. oryzae* grew as dense pellets of aggregated biomass (Figure 6). Microscopic images for the airlift cultivations, however, indicated a filamentous growth form for both of the fungi in airlift reactors (Figure 7).

### 2.6. Analysis of the Fungal Biomass and Its Derivatives

#### 2.6.1. Fungal Biomass

Fungal biomass harvested from the airlift bioreactors was analyzed for protein, lipid, and cell wall contents, *i.e.*, alkali insoluble material (AIM) (Table 2).

The fungal biomass of *M. indicus* and *R. oryzae* contained 40 and 51% protein, respectively. Total lipid contents of *M. indicus* and *R. oryzae* were 9.8 and 4.4 percent of biomass, respectively (Table 2).

#### 2.6.2. Cell Wall Analysis

Alkali insoluble materials (cell wall skeleton of biomass) yields for *M. indicus* and *R. oryzae* harvested from airlift reactors were 31% and 34%, respectively (Table 2). Analysis of cell wall ingredients is presented in Table 3. Phosphate content of *M. indicus* and *R. oryzae* cell wall was 2.7% and 2.4%, respectively. Most proteins were dissolved in alkali solution during the AIM preparation, and therefore the protein content of AIM was quite low. As can be seen in Table 3, glucosamine and *N*-acetyl glucosamine were the major constituents of AIM for both fungi. For *M. indicus*, 55% and 12% of AIM were composed of GlcN and GlcNAc, respectively. For *R. oryzae*, 59% and 17% of AIM were composed of GlcN and GlcNAc, respectively (Table 3). Therefore, total chitin-chitosan content of the cell wall was 0.60 and 0.69 *g*/*g* AIM for *M. indicus* and *R. oryzae*, respectively.

## 3. Discussion

The environmental aspects of citrus waste are challenging issues for the citrus industries. However, these materials may be considered as low-cost substrates for the production of value-added materials, e.g., by fermentation. The possibility for direct consumption of the citrus waste water extract for fungal cultivation may open up several opportunities for industrial applications in order to reduce the environmental concerns, as well as for economic improvement in these industries.

Although the two fungi demonstrated different patterns in the uptake of free sugars of CWFS and macroscopic growth, both fungi demonstrated high potentials in assimilating all glucose and most of fructose in the absence of any nutrients in the airlift reactors. For both fungi, glucose uptake happened earlier and faster than fructose uptake. This finding is according to the previous research on orange peel hydrolysates [19,20]. The same pattern in sugar uptake has been also reported for *M. indicus* under aerobic or anaerobic conditions in glucose- and fructose-containing media [17].

The microscopic images indicated more branches for *M. indicus* compared to *R. oryzae*. Besides, at this stage, it is likely for the fungus to form septa which is linked to nuclear division (Figure 6B) [14]. Nuclear division, which leads to branched hyphae, made cell propagation faster. This is a likely reason why sugar assimilation is faster. For *R. oryzae*, on the other hand, the dominant microscopic growth form happened by mainly tip extension (Figure 7E).

Filamentous fungi are morphologically characterized to grow as loose hyphal aggregates, called ‘‘mycelial clumps’’, or as denser spherical aggregates, called ‘‘pellets’’. Changing the inoculum concentration, spore viability, temperature, pH, mechanical stress, and presence or absence of seeding cultures is generally recognized as the most important parameter, affecting the fungal morphology [10,14,21]. Three mechanisms of pellet formation are (a) germination of a fraction of initial aggregated spores; (b) the noncoagulating type which a pellet originates from a single spore; (c) and the hyphal element agglomeration type [14]. As soon as the cells agglomerate together and form pellets, the mass transfer from medium to cell is severely inhibited. This caused the slower consumption rate of sugar by *R. oryzae.* Pellet formation has shown promising advantages for filamentous fungi such as improving aeration, stirring, and heat transfer. [14]. Therefore, from the practical point of view, *R. oryzae*, forming pellets, is the preferred strain for the fermentation of the free sugars in the citrus wastes. Pellet formation by *R. oryzae* in different fermentation conditions has been reported, and it was suggested that the fungus needs strong agitation to form pellets [13]. It is noteworthy to note that in shake flasks with the same conditions as the bioreactor, the fungal growth was clump mycelia, while no pellets were formed.

In this study, the fungal biomasses contained considerable amounts of protein, lipid, and chitosan. The potential of the fungus *Rhizopus oligosporus* cultured on sugar-ethanol residue, vinasse, in an airlift bioreactor (1.5 vvm) for protein production has been studied [22]. This fungal biomass (3.8 g/L) contained 50 percent protein; however, the medium was supplemented with nitrogen and phosphorus. The protein content of *M. indicus* biomass cultivated in glucose-containing medium was also reported to be 58% [23]. It was reported that by changing the morphology to yeast-like form, and inducing the fungi starvation step, that the lipid content was increased [15,24]. Regarding the fungal lipids, C_16_ and C_18_ fatty acids with oleic and linoleic are the major polyunsaturated fatty acids. These valuable fatty acids together with protein-rich biomass made the fungal biomass a suitable candidate for animal nutrition, e.g., fish feed [25].

AIM yields obtained from the cultivation of two fungi in the airlift bioreactor were considerably higher than the yields reported previously [15,26,27]. In fact, spurge aeration caused cell wall to be thicker than that in regular aeration conditions in shake flask. The AIM yields from the airlift reactor obtained were almost double than that for the filamentous-like fungi in shake flasks. Besides, the analysis of the AIMs obtained from the bioreactor showed a considerable increase in GlcN and decrease in GlcNAc content of AIM, as compared to previous findings. For *M. indicus* with filamentous-like morphology, the yields of GlcN and GlcNAc were reported as 0.48–0.50 and 0.19–0.24 *g*/*g* AIM, respectively [15]. For *R. oryzae*, the corresponding values were reported to be between 0.10 and 0.21 *g*/*g* AIM [27]. In this case, the biomass contained not only high amounts of AIM, but also considerable portions of chitosan in AIM.

## 4. Materials and Methods

Citrus wastes (CW) were obtained from Brämhults juice factory (Borås, Sweden), stored frozen at −20 °C, and used without further size reduction. The term “citrus wastes” is referred to the peel, seeds, and leaf residues remaining after juice extraction. The citrus fruits were mainly orange and grapefruits from Brazil and Spain. The CW dry weight was measured after drying a known amount of the waste for 24 h at 105 °C. Total water-soluble extractives was determined by Soxhlet apparatus [28]. The extraction solvent (water) was cycled 4 times per hour during the 24 h of extraction.

High purity liquid invertase (β-Fructofuranosidase) with approximate activity of 2000 U/mL was provided by Megazyme International Ireland Ltd., Bray, Ireland.

All other chemicals were reagent grade and purchased from Sigma-Aldrich Inc. (St. Louis, MO, USA) or Merck (Darmstadt, Germany).

### 4.1. Microorganisms

Two strains of zygomycetes fungi, *Mucor indicus* CCUG 22424 and *Rhizopus oryzae* CCUG 28958, were obtained from Culture Collection University of Göteborg, Sweden, and used in all experiments. The strains were cultivated on agar plates containing 40 g/L glucose, 10 g/L soy peptone, and 20 g/L agar, at pH 5.5 and 32 °C for 4 days.

### 4.2. Extraction of Free Sugars from CW

Citrus waste free sugars (CWFS) were extracted by mixing raw materials with distilled water and then squeezing the mixture through a fabric to obtain a clear filtrate. The mixing was done by either laboratory blender which smashed all constituents together or mixing in 1000 mL Erlenmeyer flask for 15 min at 120 rpm and room temperature. The recovery of the extraction was calculated according to the following formula:

$$\text{Recovery}=\frac{C_{\text{TS}}\times V}{W}$$

where *C*_TS_ is the total concentration of sugars (g/L), *V* is the volume of filtrate (L), and *W* is the initial weight of CW (kg). 

The filtrate was either used directly or supplemented with nutrients to a final concentration of 5 g/L yeast extract, 7.5 g/L (NH_4_)_2_SO_4_, 3.5 g/L KH_2_PO_4_, 0.75 g/L MgSO_4_·7H_2_O, and 1.0 g/L CaCl_2_·2H_2_O.

### 4.3. Cultivation of Fungi in Shake Flasks

The fungal spores were harvested aseptically by pouring 10 mL sterile distilled water to the plates and gently agitating the mycelia. Shake flask cultivations were performed in 1000 mL baffled flasks at 120 rpm and 30 °C and 35 °C for *M. indicus* and *R. oryzae*, respectively. The fungal spore suspension, 2% (*v*/*v*), was added to 250 mL of the sterile CW water soluble extract (with or without supplementary nutrients). The medium pH was adjusted to 5.5 by sodium hydroxide (2 M). Samples were taken aseptically at regular time intervals and analyzed for sugar and ethanol contents. At the end of fermentation (48 h and 72 cultivation for *M. indicus* and *R. oryzae*, respectively), the mycelia were harvested by centrifugation (10 min, 9000× *g*) and washed with water before freeze-drying.

### 4.4. Cultivation of Fungi in Airlift Reactor

Airlift operating conditions were set at 3.5 L working volume, pH 5.50 (adjusted by 2 M sodium hydroxide solution), 30.0 °C (*M. indicus*) or 35.0 °C (*R. oryzae*), and 0.9 vvm (volume of air per volume of reactor per min) aeration rate (through a stainless steel sparger with 90 micron pore size). The reactor pattern flow was riser and downcomer type. The inlet air was passed through a 0.2 μm pores sterile polytetrafluoroethylene filter and entered the bottom of the reactor. An amount of 100 μL invertase, to convert sucrose to glucose and fructose, and 2% (*v/v*) spore inoculation were added to the sterile airlift medium at the beginning of cultivation. Cultivation was performed for 48 and 72 h for *M. indicus* and *R. oryzae*, respectively. Liquid samples were periodically collected from the culture during the cultivation, centrifuged, and analyzed for soluble sugars and ethanol contents.

At the end of each cultivation, the fungal biomass was harvested by filtration through a metal sieve, the filtrate was discarded, and the mycelia were washed with water until a clear filtrate observed. The biomass was then freeze-dried and stored at −20 °C for further analysis of lipid, protein, and cell wall components. The dried solids were measured gravimetrically, and the biomass yield was calculated based on grams of dried biomass produced per gram of consumed sugars.

### 4.5. Fungal Biomass Analysis

#### 4.5.1. Lipid

Total lipid was extracted by the modified method presented by Folch *et al.* [29]. Briefly, 100 mg of lyophilized biomass was dispersed in 20 mL of 1:2 (*v*/*v*) chloroform-methanol and sonicated at 35 kHz for 3 min. An ultrasonic bath (Elmasonic P, Singen, Germany) at 400 W containing 1 L of distilled water was used for the treatments. To prevent temperature rising during the sonication, ice cubes were added to the water bath. The suspension was then gently agitated at room temperature for 2 h. After centrifuging at 8700 rpm for 5 min, 10 mL of upper oil-containing phase was transferred to 4 mL distilled water to impart a phase separation. The lower organic phase was dried under fume hood overnight, and the total lipid weight was determined gravimetrically.

#### 4.5.2. Protein

Protein content of fungal biomass was determined by biuret method [30]. An amount of 10 mg of sample was mixed with 3 mL of 1 M sodium hydroxide and boiled for 10 min. After immediate cooling in an ice bath, 1 mL of 2.5% CuSO_4_·5H_2_O was added to the test tubes and mixed gently for 5 min. The clear supernatant was collected after centrifuging, and the absorbance was read against distilled water at 555 nm. Different concentrations of bovine serum albumin (BSA) in 1 M sodium hydroxide were used as standards.

#### 4.5.3. Cell Wall Analysis

The dried biomass was treated with 0.5 M sodium hydroxide (30 mL per g dried biomass) and autoclaved at 121 °C for 20 min to remove proteins, lipids, and other alkali soluble fractions. Alkali insoluble material (AIM), as a representative for fungal cell wall, was separated by centrifuging at 9900× *g* for 10 min, washed 10 times with deionized water, freeze-dried, and weighed. Afterwards, 10 mg of AIM was subjected to a two-step sulfuric-acid hydrolysis followed by one step nitrous acid treatment to hydrolyze the chitosan and chitin fractions of AIM into 2,5-anhydromannose and acetic acid. Glucosamine (GlcN) and *N*-acetyl glucosamine (GlcNAc) contents of AIM were determined by measuring the concentration of 2,5-anhydromannose and acetic acid, respectively. The concentration of 2,5-anhydromannose and acetic acid was detected by high performance liquid chromatography [26].

After dilute sulfuric acid treatment, 500 μL of hydrolyzate was taken to analyze the phosphate content of the cell wall according to European standard ISO6878 method [31]. Phosphate ion was reacted with acid molybdate reagent in the presence of ascorbic acid solution to form a light blue color. Phosphate concentration was determined spectrometrically at 880 nm. Potassium dihydrogen phosphate was used as a standard.

### 4.6. Analytical Methods

A high performance liquid chromatography (HPLC, Jasco International Co., Tokyo, Japan) with an ion-exchange Aminex column (HPX-87H, Bio-Rad, Richmond, CA, USA) was used to analyze ethanol, acetic acid, and 2,5-anhydromannose at 60 °C with 0.6 mL/min eluent of 5 mM sulfuric acid. Ethanol and 2,5-anhydromannose were detected with RI detector while acetic acid was detected using a UV-Vis detector.

Sucrose, glucose, and fructose were measured by the assay kit Megazyme K-SUFRG (Megazyme, Bray, Ireland). The samples were diluted 50 times and filtered through 0.2 μm syringe filter before analyses.

## 5. Conclusions

Fermentation of citrus waste free sugars without auxiliary nutrients by zygomycetes fungi provides the advantages of reducing wastes and producing valuable chemicals. The fungal biomass obtained from cultivation in the airlift bioreactor contained considerable amounts of chitosan, fungal oil, and protein. Pellet formation by *R. oryzae* has shown many advantages, e.g., lowering broth viscosity and facilitating aeration, which made it suitable for industrial applications.

## Figures and Tables

**Table 1:** Extraction of citrus waste free sugars (CWFS) at room temperature.

Number	Raw CW (g)	Water Added (mL)	Mixing Condition	Final Liquid Volume (mL)	Recovery (g sugars/kg raw CW)
1	250	250	Mixer	170	19.7
2	150	250	Mixer	250	29.7
3	250	250	Shaking at 120 rpm (15 min)	250	24.8
4	150	250	Shaking at 120 rpm (15 min)	250	30.3

**Table 2:** Analysis of fungal biomass obtained from cultivation of the fungi on CWFS in airlift bioreactor without any auxiliary nutrients. Data are mean ± SD and *n* = 3.

Component	*M. indicus*	*R. oryzae*
Biomass (g/L)	4.7 ± 0.2	4.0 ± 0.1
Biomass yield (*g*/*g* sugars consumed)	0.25 ± 0.01	0.28 ± 0.00
Protein ^1^	0.40 ± 0.01	0.51 ± 0.07
Total lipids ^1^	0.098 ± 0.011	0.044 ± 0.007
AIM ^2^ yield ^1^	0.31 ± 0.01	0.34 ± 0.01

^1^ *g*/*g* of biomass; ^2^ Alkali insoluble materials.

**Table 3:** Cell wall analysis of fungal biomass obtained from airlift bioreactor without any auxiliary nutrients. Data are mean ±SD and *n* = at least 2.

Cell Wall Derivatives	*M. indicus*	*R. oryzae*
Phosphate ^1^	0.027 ± 0.003	0.024 ± 0.006
GlcN ^1^	0.551 ± 0.048	0.596 ± 0.018
GlcNAc ^1^	0.121 ± 0.041	0.168 ± 0.041
Protein ^1^	0.054 ± 0.001	0.076 ± 0.004

^1^ *g*/*g* of AIM (alkali insoluble materials).

## Abbreviations

AIM	Alkali insoluble materials
CW	Citrus wastes
CWFS	Citrus waste free sugars
GlcN	Glucosamine
GlcNAc	*N*-acetyl glucosamine
